# Torque for an Inertial Piezoelectric Rotary Motor

**DOI:** 10.1155/2013/854126

**Published:** 2013-12-28

**Authors:** Jichun Xing, Lizhong Xu

**Affiliations:** Mechanical Engineering Institute, Yanshan University, Qinhuangdao 066004, China

## Abstract

For a novel inertial piezoelectric rotary motor, the equation of the strain energy in the piezoceramic bimorph and the equations of the strain energy and the kinetic energy in the rotor are given. Based on them, the dynamic equation of the motor is obtained. Using these equations, the inertial driving torque of the motor is investigated. The results show that the impulsive driving torque changes with changing peak voltage of the excitation signal, the piezoelectric stress constant, the thickness of the piezoceramic bimorph, and the rotor radius obviously. Tests about the motor torque are completed which verifies the theory analysis here in. The results can be used to design the operating performance of the motor.

## 1. Introduction

Piezoelectric motors maintain relatively high torque at relatively low speeds, without a reduction gear [1, 2]. Among all of the piezoelectric motors, inertial drive principle has the advantage of simpler requirements to the construction and driving circuitry [3].

One type of the inertial motors is based on the impact drive mechanism from impulse inertial force [4]. Using the mechanism, the micromanipulator for cell manipulation and auxiliary positioning system for STM and AFM were developed [5, 6]. Another type of the inertial motors is based on the smooth impact drive mechanism. Here, a base plate or bar is driven with rapid expansion and slow shrinkage. The slider on the base slips during rapid motion and follows the base due to frictional force. With this principle, many applications were proposed and fabricated [7–9]. Choi et al. proposed a dynamic model to investigate dynamic characteristics of a novel type of inertial actuator and verified the model through comparison of voltage-dependent actuating forces between experiment and analysis [10]. Lipanov et al. proposed an inertial piezoelectric step drive for subnanosize-accuracy movements [11]. Gulyaev et al. used backlash-free screw-nut pairs in an inertial piezodrive to increase movement accuracy of an inertial piezoelectric drive [12]. Mazeika and Vasiljev proposed a novel design of tiny piezoelectric inertial motor based on inertial motion of the slider applying sticks and slip phases between stator and slider, and a prototype inertial piezoelectric motor was built. The motor has simple design and consists of the slider with a bimorph piezoceramic disc and the clamped cylindrical shaft used for sliding [13].

However, these motors belong to the linear inertial piezoelectric motors and the inertial piezoelectric rotary motor was seldom reported. For further miniaturization of the rotational piezoelectric motors, the authors proposed a novel inertial piezoelectric rotary motor [14]. The motor structure is simple and further miniaturization of the rotational piezoelectric motor can be done easily.

However, the inertial driving torque of the motor has not been investigated yet. It is unfavorable to design the load-carrying ability of the motor.

In this paper, the equations of the strain energy in the piezoceramic bimorph and the equations of the strain energy and the kinetic energy in the rotor are deduced. Based on them, the dynamic equation of the motor is obtained. Using these equations, the inertial driving torque of the motor is investigated. Tests about the relationship between the motor torque and the exciting voltage frequency or amplitude are done. The results are useful for design and control of the operating performance of the motor.

## 2. Voltage Excitation

**Figure 1:**
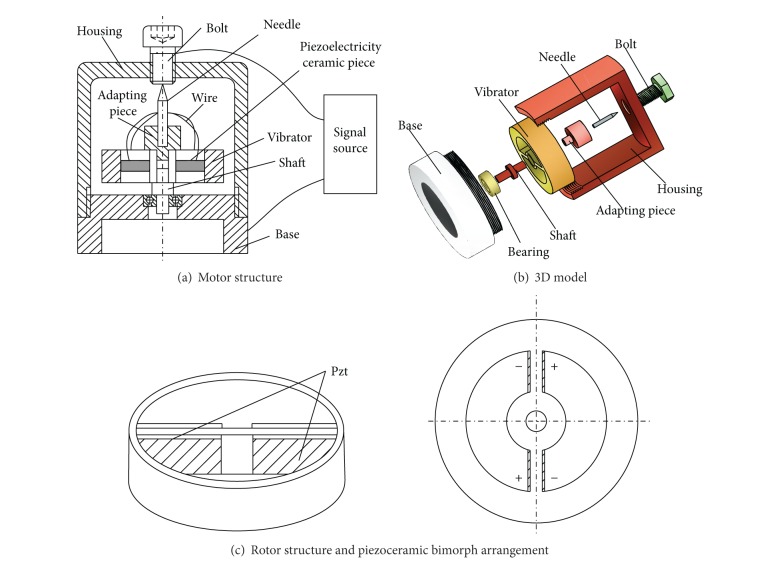
Motor and rotor structures.

As shown in Figure 1, the novel inertial rotary motor consists of a stator and a rotor. The rotor includes an outer ring, an inner ring, and two ribs connecting the outer ring to the inner ring. The inner ring is mounted on a supporting bearing and the outer ring is used as the inertial mass. The piezoceramic bimorph is adhered on each side surface of the two ribs. As soon as a rapid rise input voltage is supplied to the motor, it excites the transverse bending vibration of the two ribs. Thus, the inertial force within the outer ring occurs which causes inertial torque to be applied to the rotor and makes it rotate. Then, a slow decreasing input voltage is supplied to the motor, so the inertial forces within the outer ring are so small that the inertial torque can be balanced by the friction torque between the rotor and the bearing. Thus, the rotor does not rotate. The rapidly increasing and slowly decreasing input voltage with a special frequency is supplied to the motor periodically, and then the rotor can rotate continuously.

**Figure 2:**
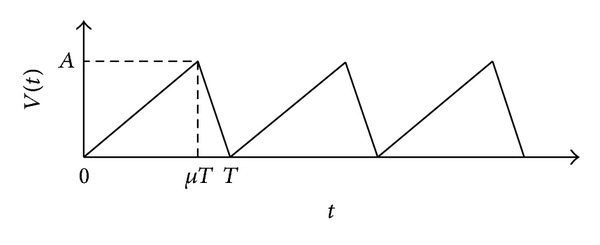
Applied electrical signal wave.

For the motor motion, a saw-tooth-type electric signal is applied (see Figure 2). Here, *t* is the time, *T* is the period of the saw-tooth cycle, *μ* (0 < *μ* < 1) is the ratio of the rise time to the period, and *A* is the amplitude of voltage signal *V*(*t*). The voltage signal can be written by

(1) V(t)={AμTt(0≤t<μT)A(μ−1)Tt+A1−μ(μT≤t≤T).

The voltage signal can be written in the Fourier series as

(2) $$\begin{array}{c} V\left(t\right)=a_{0}+\sum_{n=1}^{\infty }\left[a_{n}\cos\left(n\omega t\right)+b_{n}\sin\left(n\omega t\right)\right], \end{array}$$

where

(3) a0=1T∫0TV(t)dt=1T(∫0μTAμTtdt+∫μTT(A(μ−1)Tt+A1−μ)dt)=12Aan=2T∫0TV(t)cos⁡(nωt)dt=A(−1+cos⁡(2nμπ))2n2π2μ(1−μ),bn=2T∫0TV(t)sin(nωt)dt=Asin(2nμπ)2n2π2μ(1−μ).

## 3. The Impulsive Moment

**Figure 3:**
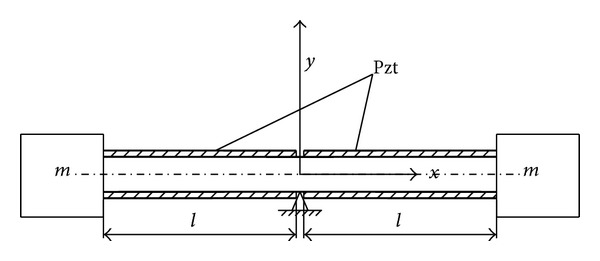
Dynamics model of the rotor.

The rotor of the motor can be considered as a beam with inertial mass at its two ends. Four piezoceramic bimorphs are adhered on side surface of the beam (see Figure 3). The strain in the piezoceramic bimorphs can be calculated as

(4) $$\begin{array}{c} \varepsilon_{1}=\frac{h}{2}\frac{\partial^{2}y\left(x,t\right)}{\partial x^{2}}, \end{array}$$

where *y*(*x*, *t*) is the transverse displacement of the beam, *x* is the length coordinate of the beam, *h* is the thickness of the beam, and *ε*_1_ is the strain in the piezoceramic bimorphs.

By substituting (4) into the piezoelectric equation, the stress in the piezoceramic bimorph can be given [15] as

(5) $$\begin{array}{c} \sigma_{1}\left(x,t\right)=-e_{31}E_{3}+\frac{h}{2}c_{11}^{E}\frac{\partial^{2}y\left(x,t\right)}{\partial x^{2}}, \end{array}$$

where *E*_3_ is the electric-field intensity on the piezoceramic bimorph, *E*_3_ = *V*(*t*)/*h*_*p*_, *h*_*p*_ is the thickness of the piezoceramic bimorph, *e*_31_ is the piezoelectric stress constant, *c*_11_^*E*^ is the stiffness constant, and *σ*_1_(*x*, *t*) is the stress in the piezoceramic bimorph.

From (4) and (5), we know that the strain energy *V*_*p*_ in the piezoceramic bimorph is

(6) Vp=b2∫0lpσ1(x,t)ε1dx=bh4∑i=1∞qi(t)∫0lp−e31E3ϕ′′(x)dx+12∑i=1∞kijpqi(t)qj(t),

where *y*(*x*, *t*) = ∑_*n*=1_^*∞*^ *ϕ*_*n*_(*x*)*q*_*n*_(*t*), *k*_*ij*_^*p*^ = *k*_*ji*_^*p*^ = ∫_0_^*l*^*p*^^(*bh* ^2^/4)*c*_11_^*E*^ *ϕ*_*i*_′′(*x*)*ϕ*_*j*_′′(*x*)*dx*, *ϕ*_*j*_(*x*) is the mode function, and *l*_*p*_ and *b* are the length and width of the piezoceramic bimorph, respectively.

The strain energy *V*_*L*/*P*_ in the rotor beam is

(7) $$\begin{array}{c} V_{L/P}=\frac{1}{2}\int_{0}^{l}EI{\left[\frac{\partial^{2}y\left(x,t\right)}{\partial x^{2}}\right]}^{2}dx=\frac{1}{2}\sum_{i=1}^{\infty }\;\sum_{j=1}^{\infty }k_{ij}^{l}q_{i}\left(t\right)q_{j}\left(t\right), \end{array}$$

where *k*_*ij*_^*l*^ = *k*_*ji*_^*l*^ = ∫_−*l*_^*l*^ *EIϕ*_*i*_′′(*x*)*ϕ*_*j*_′′(*x*)*dx* and *l* is the length of the rotor beam (see Figure 3).

Four piezoceramic bimorphs are used in the motor, and then the total strain energy *V* in the rotor and the piezoceramic bimorphs is

(8) $$\begin{array}{c} V=4V_{p}+V_{L\setminus P}. \end{array}$$

Kinetic energy *E*_*m*_ of the rotor is

(9) $$\begin{array}{c} E_{m}=\frac{1}{2}\int_{-l}^{l}\rho S{\left[\frac{\partial y}{\partial t}\left(x,t\right)\right]}^{2}dx=\frac{1}{2}\sum_{i=1}^{\infty }\;\sum_{j=1}^{\infty }m_{ij}{\dot{q}}_{i}\left(t\right){\dot{q}}_{j}\left(t\right), \end{array}$$

where *m*_*ij*_ = *m*_*ji*_ = ∫_−*l*_^*l*^ *ρSϕ*_*i*_(*x*)*ϕ*_*j*_(*x*)*dx* + *mϕ*_*i*_(−*l*)*ϕ*_*j*_(−*l*) + *mϕ*_*i*_(*l*)*ϕ*_*j*_(*l*) is the equivalent mass of the rotor and *m* is the inertial mass.

Substituting (8) and (9) into Lagrange equation yields

(10) $$\begin{array}{c} M\ddot{q}\left(t\right)+C\dot{q}\left(t\right)+K=F\left(t\right), \end{array}$$

where **M** = [*m*_*ij*_] is the mass matrix, **K** = [*k*_*ij*_] is the stiffness matrix, *k*_*ij*_ = *k*_*ij*_^*p*^ + *k*_*ij*_^*l*^, **F**(*t*) is the generalized force vector, **C** = [*c*_*ij*_] is the damping matrix, and *c*_*i*__*j*_ = *c*_*ji*_ = ∫_0_^*l*^ *C*_*d*_ *ϕ*_*i*_(*x*)*ϕ*_*j*_(*x*)*dx*; here the friction damping between stator and rotor is considered and the equivalent damping coefficients are determined from equal energy principle.

Using the orthogonality of the mode functions, (10) can be changed into the following form uncoupled to each other:

(11) $$\begin{array}{c} M_{n}\ddot{q}\left(t\right)+C_{n}\dot{q}\left(t\right)+K_{n}q\left(t\right)=F_{n}\left(t\right), \end{array}$$

where **M**_**n**_, **C**_**n**_, and **K**_**n**_ are the diagonal mass, damping, and stiffness matrixes. $F_{n}\left(t\right)={\left\{\begin{array}{cccc} F_{1}\left(t\right) & \cdots & F_{i}\left(t\right) & \cdots \end{array}\right\}}^{T}$ is the regular force vector.

Each element *F*_*i*_(*t*) of the regular force vector is

(12) Fi(t)=bh4(2∫−lle31E3δ(x−xa)ϕi′′(x)dx+2∫−lle31E3δ(x−xb)ϕi′′(x)dx)=bhe31V2hp(ϕi′′(xa)+ϕi′′(xb)),

where *x*_*a*_ and *x*_*b*_ are the average positions of the piezoceramic bimorphs.

The solution of (11) is

(13) $$\begin{array}{c} q_{j}=\frac{1}{M_{j}\omega_{rj}}\int_{0}^{t}F_{j}\left(\tau \right)e^{-\xi_{j}\omega_{j}(t-\tau )}\sin\omega_{rj}\left(t-\tau \right)d\tau . \end{array}$$

By substituting (2) and (12) into (13) and neglecting the transient solution, the steady solution can be obtained

(14) qj=FnMjωj2{a0+∑n=1∞1(1−γnj2)2+(2ξjγnj)2×[ancos⁡(nωt−φnj)+bnsin(nωt−φnj)]}.

Here, *φ*_*nj*_ = arctan⁡(2*ξ*_*j*_ *γ*_*nj*_/(1 − *γ*_*nj*_^2^)).

The steady response of the rotor to electric excitation is

(15) y(x,t)=∑j=1∞ϕj(x)qj(t)=∑j=1∞Fnϕj(x)Mjωj2 ×{a0+∑n=1∞an2+bn2(1−γnj2)2+(2ξjγnj)2×cos⁡(nωt−φnj−arctan(bnan))}.

From (15), the velocity $\dot{y}\left(x,t\right)$ and acceleration $\ddot{y}\left(x,t\right)$ of the motor rotor can be given.

From $\ddot{y}\left(x,t\right)$ and *T*(*t*) = −*J*_*h*_ *α*, the impulsive moment for the motor can be given as

(16) T(t)=∑j=1∞JhFnϕj(l)lMjωj2∑n=1∞n2ω2(1−γnj2)2+(2ξjγnj)2×[ancos⁡(nωt−φnj)+bnsin(nωt−φnj)],

where *J*_*h*_ is the rotary inertia of the rotor, *α* is the angular acceleration of the rotor end, and $\alpha =\ddot{y}\left(x,t\right)/l$.

## 4. Simulation and Test

**Figure 4:**
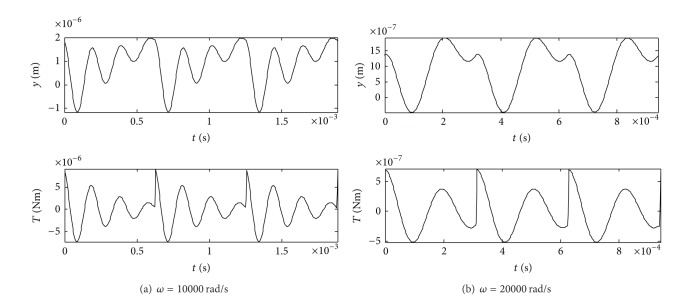
The displacement response and the corresponding impulsive moment.

Equations in this paper are utilized for the impulsive moment analysis of the inertial piezoelectric rotary motor. The parameters of the numerical example are shown in Table 1. The displacement response of the rotor end to the excitation signals and the corresponding impulsive moment under the different exciting frequencies are given in Figure 4 (here, *ω* is the frequency of the excitation signals). From Figure 4, the following observations were worth noting.

1. Under the periodic voltage excitation with saw-tooth wave, the dynamic displacement at the end of the rotor changes periodically. The rise edge of the excitation voltage corresponds to multipeaks of the dynamic displacement which are different from each other, and its trailing edge corresponds to a large peak in an opposite direction.
2. Under the periodic voltage excitation with saw-tooth wave, the changes of the impulsive moment along with time are similar to those of the dynamic displacement at the end of the rotor. As the frequency of the excitation signal grows, the peak number of the impulsive moment drops for the rise edge of the excitation voltage.
3. The driving torque of the motor is equal to the integral of the impulsive moment at a given time range. The results show that the integral is not zero which means that a driving torque is produced.
4. At a given time range, the integral of the impulsive moment is different for the different voltage excitation frequency. So, the changes of the driving torque of the motor along with the voltage excitation frequency should be investigated further.

**Figure 5:**
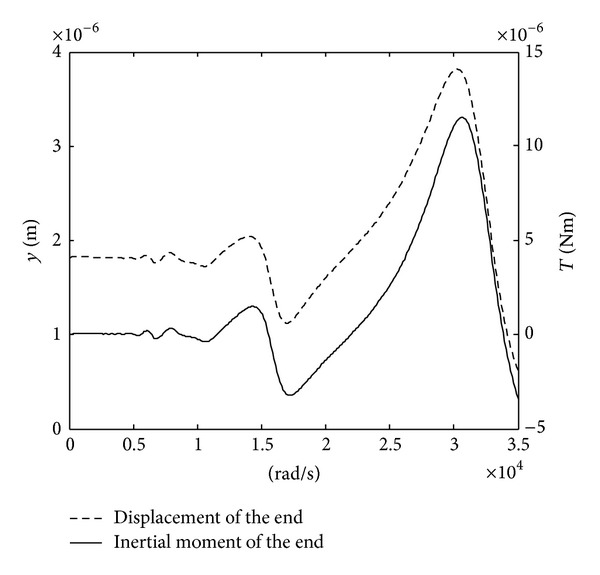
The frequency response of the displacement and impulsive moment.

The frequency responses of the displacement at the rotor end and the corresponding impulsive moment are given in Figure 5 (here, *μ* = 0.7 and *ξ* = 0.1). Figure 5 shows the following.

1. As the frequency of the voltage excitation is near one-nth of the natural frequency, the peaks of the displacement at the rotor end and the corresponding impulsive moment occur.
2. As the frequency of the voltage excitation is near the natural frequency of the motor (*n* = 1), the peaks of both displacement at the rotor end and the corresponding impulsive moment are the maximum.
3. As the frequency of the voltage excitation is equal to one-nth of the natural frequency, the jumping from positive peak to negative peak occurs. This is not favorable to operation of the motor. The frequency of the voltage excitation should be taken as slightly smaller than one-nth of the natural frequency.

**Figure 6:**
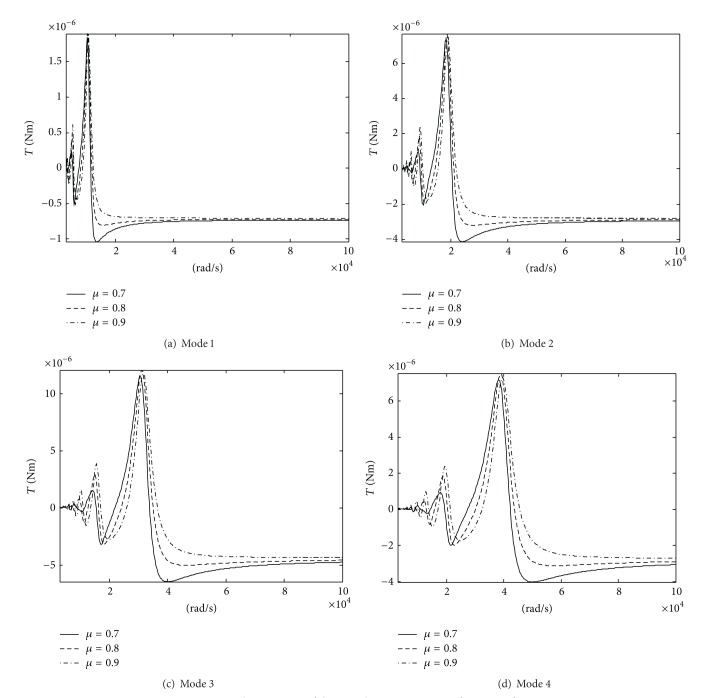
The response of the impulsive moment as a function of *μ*.

Figure 6 shows the impulsive moment as a function of the ratio of the rise time to the period for the excitation voltage. It shows the following.

1. As the ratio of the rise time to the period for the excitation voltage grows, the peak of the impulsive moment grows. The effects of the *μ* value on the positive impulsive moment peak are relatively small, and its effects on the negative impulsive moment peak are relatively large.
2. For the different modes, the impulsive moment peaks are different from each other at the same *μ*. For mode 3, the impulsive moment peak is the maximum. It shows that mode 3 is the most favorable for the motor operation.

**Figure 7:**
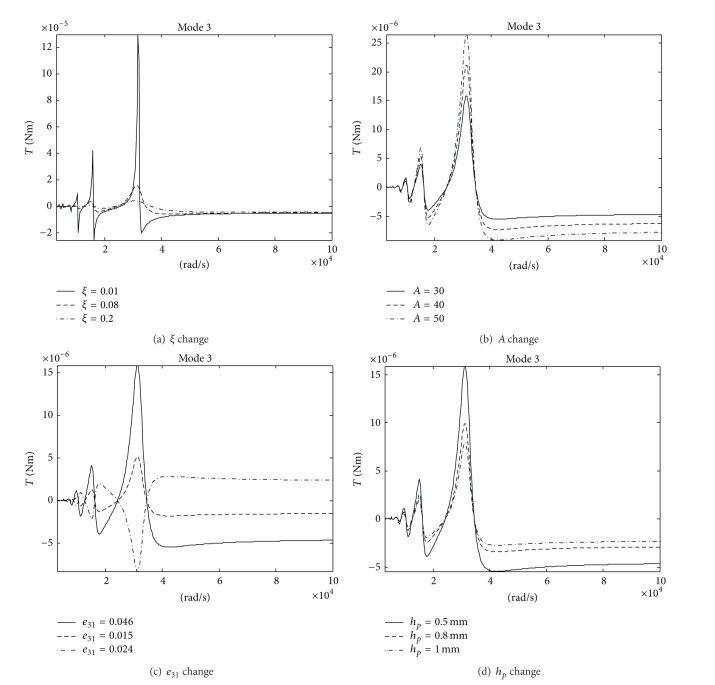
Changes of the impulsive moment along with the system parameters.

**Figure 8:**
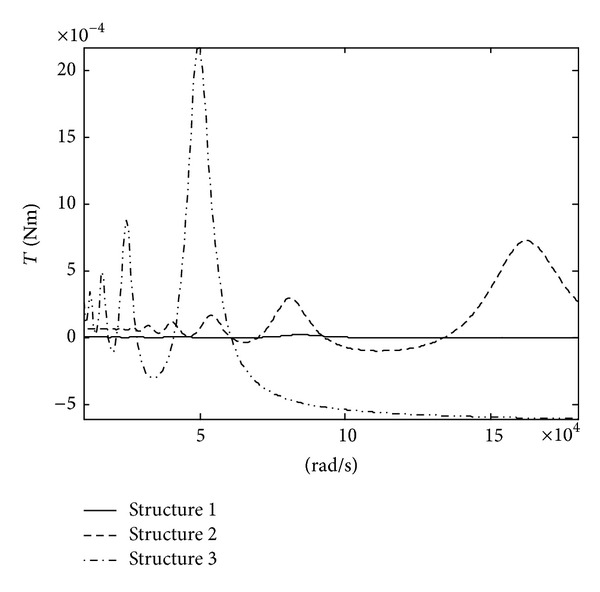
Changes of the impulsive moment along with the motor sizes.

Changes of the impulsive moment along with the system parameters are investigated (see Figure 7). Changes of the impulsive moment along with the motor sizes are given in Figure 8.  Table 2 shows three different motor sizes and corresponding exciting frequencies. Here, only the results for mode 3 are given. They show the following.

1. As the damping coefficient *ξ* drops, the impulsive moment of the motor grows. When the damping coefficient *ξ* is reduced to one-eighth, the impulsive moment is increased by more than 6 times. So, the small damping should be maintained to increase the driving torque of the motor.
2. As the peak voltage *A* of the excitation signal grows, the impulsive moment of the motor grows. The impulsive moment increases nearly linearly with increasing the peak voltage *A*.
3. As the piezoelectric stress constant *e*_31_ grows, the impulsive moment of the motor grows. The impulsive moment increases nearly linearly with increasing the piezoelectric stress constant *e*_31_ as well. So, the piezoelectric material with larger constant *e*_31_ should be selected.
4. As the thickness *h*_*p*_ of the piezoceramic bimorph grows, the impulsive moment of the motor grows. So, larger thickness *h*_*p*_ of the piezoceramic bimorph should be taken.
5. As the motor size grows, the impulsive moment of the motor grows obviously. It is because the rotary inertia of the motor grows obviously with increasing the motor size. Hence, the design of the motor size should be done according to load-carrying requirement.

**Figure 9:**
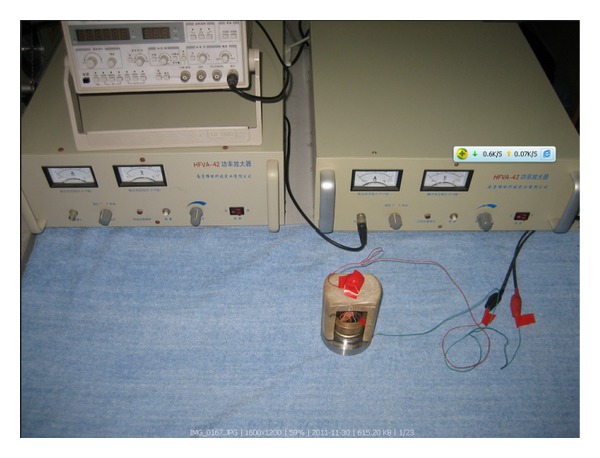
The prototype motor and its driving system.

**Figure 10:**
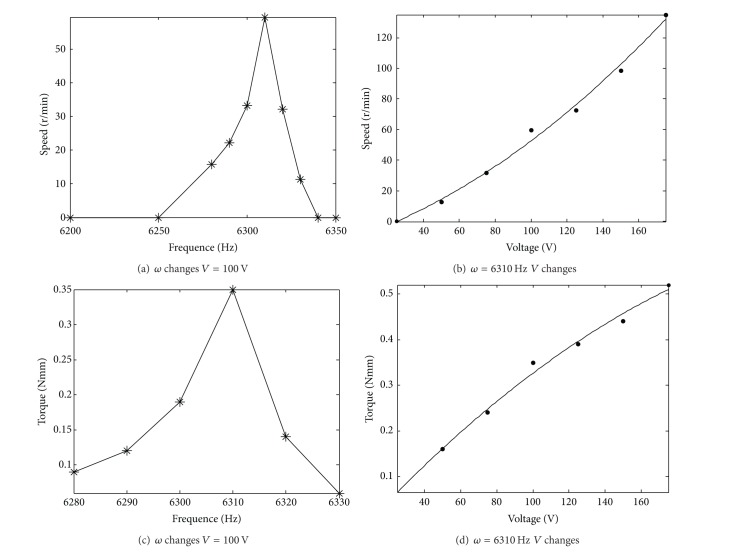
Changes of the motor speed and torque along with exciting frequency and voltage.

Tests are done on the inertial rotary motor to obtain the relationship between the motor torque or speed and the exciting voltage frequency or amplitude. The motor was driven by a signal generator (YB-1602) and a power amplifier (HFVA-42). Here, the saw-tooth wave voltage signals were used (*μ* = 0.9). The prototype motor and its driving system are given in Figure 9. The experimental results are given in Figure 10. The comparison between the experimental results and the calculated values is given in Table 3. They show the following.

1. As the peak of the voltage signal is 100 V, the model motor starts to rotate at *ω* = 6280 Hz. As the exciting frequency grows, the rotating speed of the motor increases significantly. It gets to the maximum value (59.6 rpm) at *ω* = 6310 Hz. As the exciting frequency further grows, the rotating speed of the motor decreases quickly. It stops to rotate at *ω* = 6330 Hz.
2. For *ω* = 6310 Hz, the motor starts to rotate at *V* = 25 V. The rotating speed of the motor grows nearly linearly with increasing the amplitude of the exciting voltage (from 25 V to 170 V).
3. The above results are obtained under the condition without the outer load. Here, the motor only bears the friction force within the motor. Under the outer load and *V* = 100 V, the output torque of the motor increases significantly with increasing the exciting frequency. It gets to the maximum value (0.35 Nmm) at *ω* = 6310 Hz. As the exciting frequency further grows, the torque of the motor decreases quickly. The torque is zero at *ω* = 6330 Hz.
4. For *ω* = 6310 Hz, The output torque of the motor grows obviously with increasing the amplitude of the exciting voltage. At *V* = 175 V, the output torque of the motor is 0.52 Mmm.
5. For *ω* = 6310 Hz and *V* = 75–175 V, the relative errors between the measured torque and the calculated average torque are about 30–40%. It shows that the theory analysis results in this paper are believable.

## 5. Conclusions

In this paper, the dynamic equation of the inertial piezoelectric rotary motor is obtained. The inertial driving torque of the motor is analyzed and tested. The results show the following.

1. The jumping from positive peak to negative peak occurs when the frequency of the voltage excitation is equal to the natural frequency which is not favorable for the motor. So, the frequency of the voltage excitation should be taken as slightly smaller than the natural frequency.
2. The impulsive driving torque changes with changing the system parameters. Larger peak voltage of the excitation signal, larger piezoelectric stress constant, larger thickness of the piezoceramic bimorph, and larger radius of the rotor can give larger impulsive driving torque.
3. The experimental output torque verifies the theory analysis here in.

## Figures and Tables

**Table 1:** Parameters of the numerical example.

*R*_1_ (mm)	*R*_2_ (mm)	*h* (mm)	*l* (mm)	*E* (Gpa)
12	13	1	12	120

*l*_*p*_ (mm)	*b* (mm)	*h*_*p*_ (mm)	*e*_31_ (mm)	*ρ* (kg·m^−3^)

10	5	0.5	−0.046	8.9 × 10^3^

**Table 2:** Different motor sizes and exciting frequencies.

Size number	*R*_1_ (mm)	*R*_2_ (mm)	*k* (mm)	*l* (mm)	*h* (mm)	*ω* (rad/s)
1	13	12	5	12	1	84254
2	18	15	10	15	2	161768
3	25	17.5	15	17.5	3	49336

**Table 3:** Comparison of experimental results and theoretical values.

Signal voltage (V)	Calculated maximum torque (Nmm)	Calculated average torque (Nmm)	Measured torque (Nmm)	Relative errors (%)
75	0.98	0.37	0.24	35
100	1.31	0.50	0.35	30
125	1.7	0.62	0.39	37
150	2.01	0.75	0.44	41
175	2.3	0.87	0.52	40
